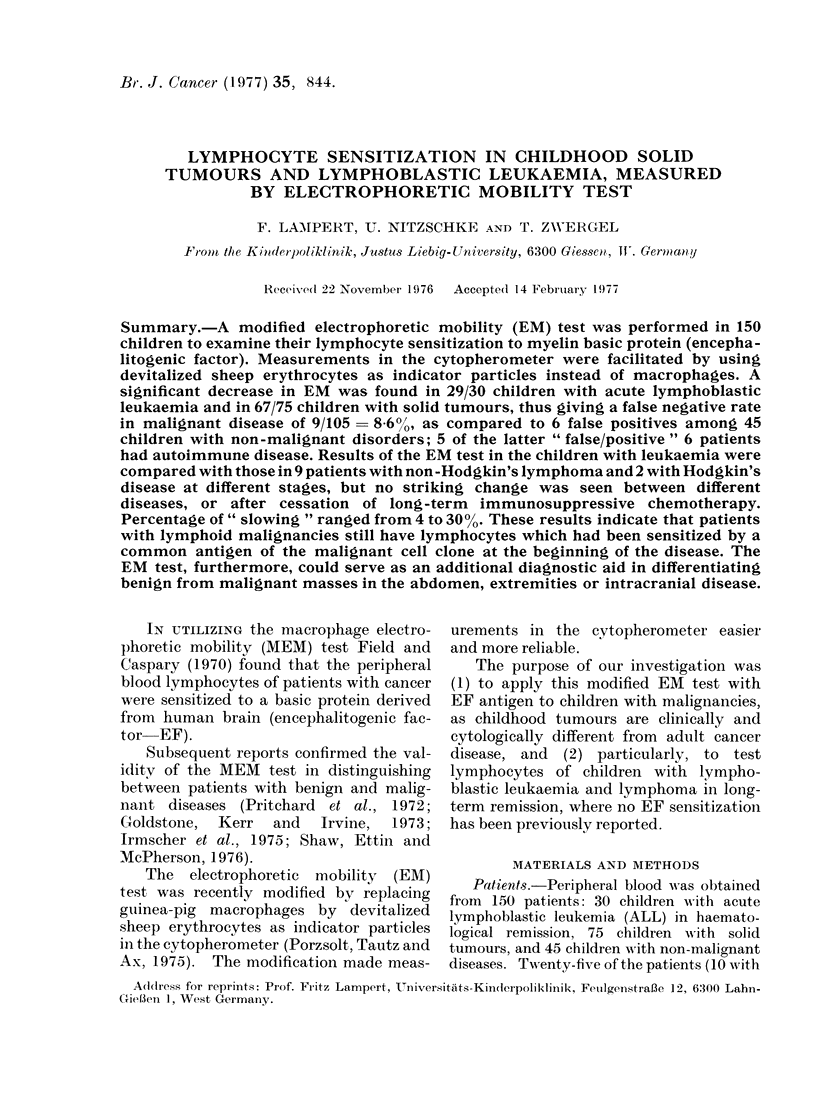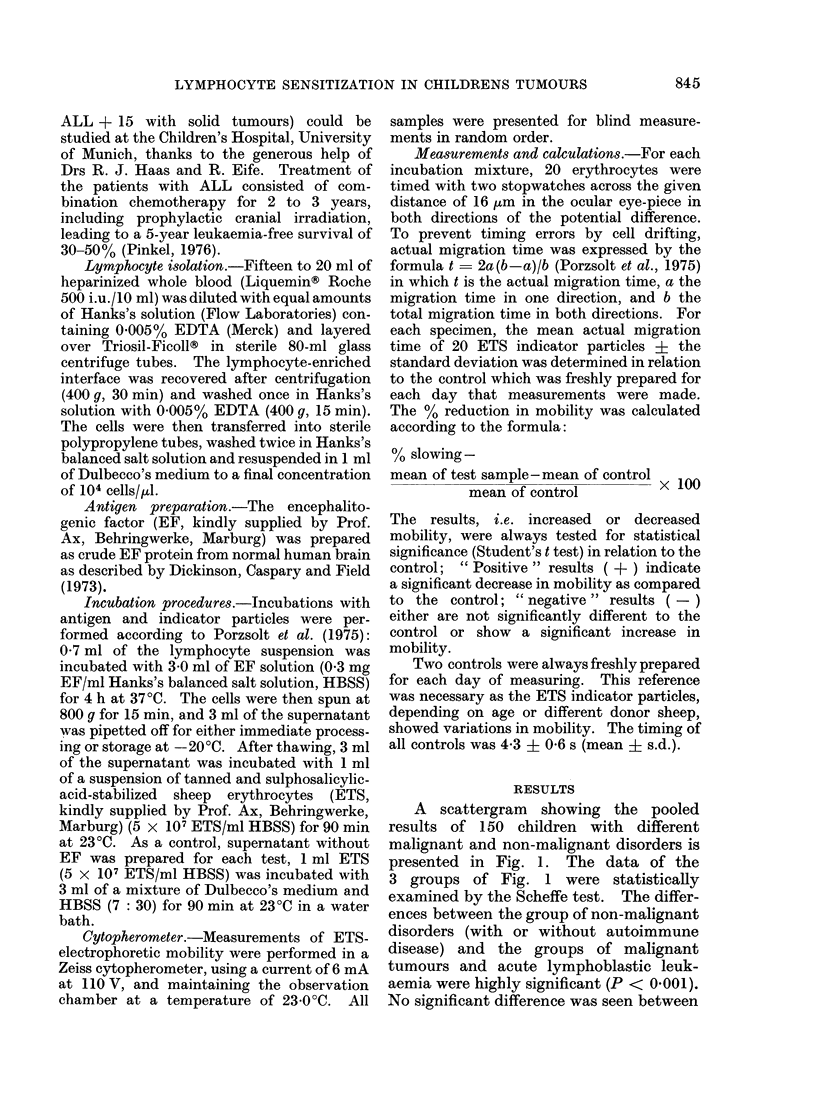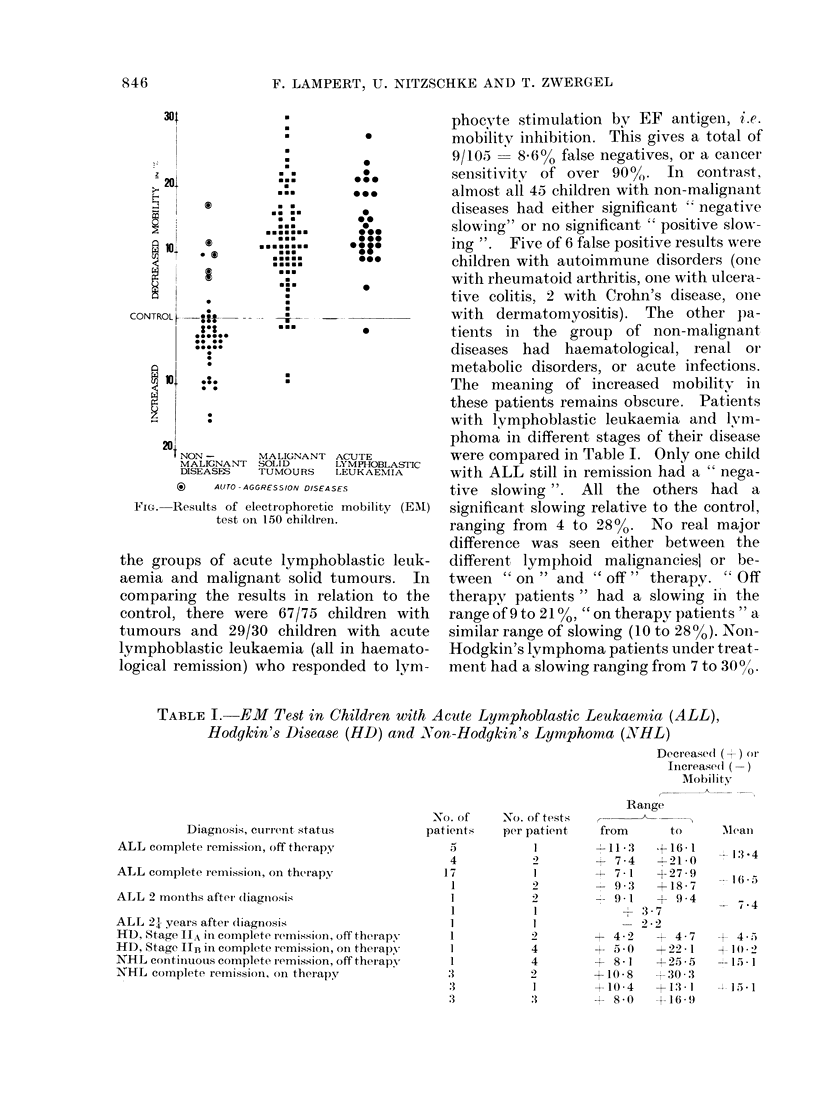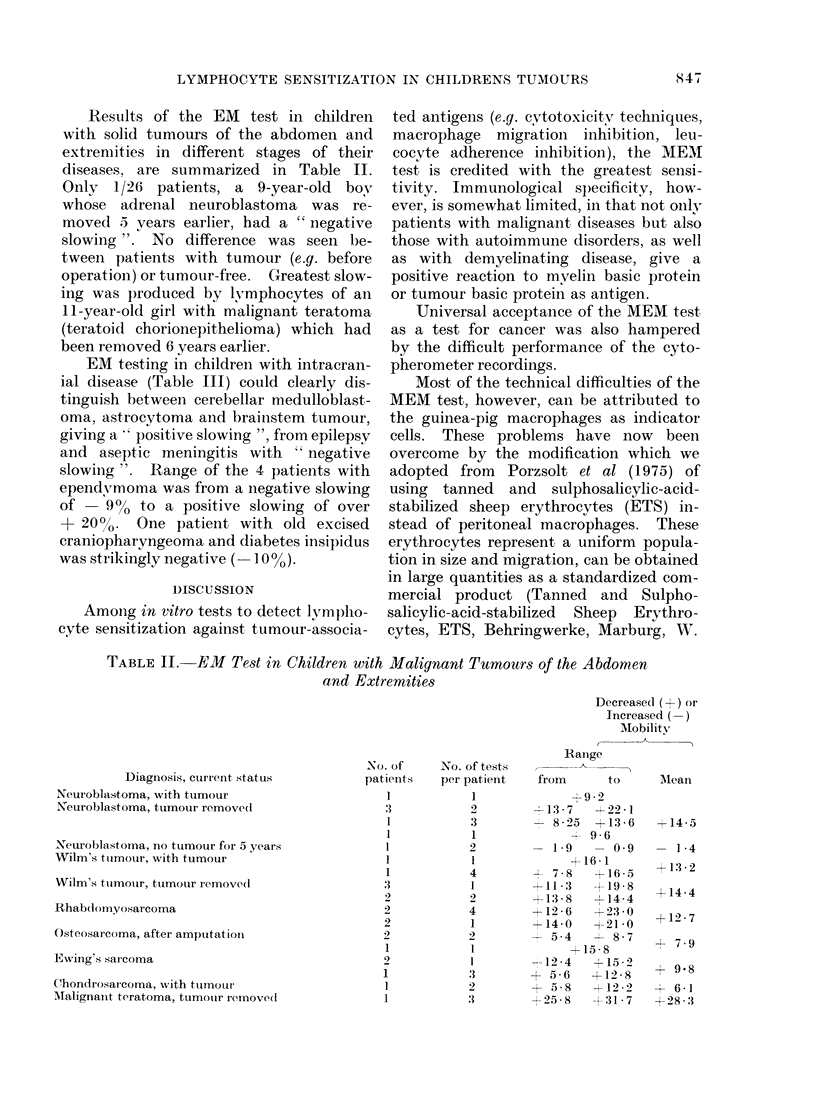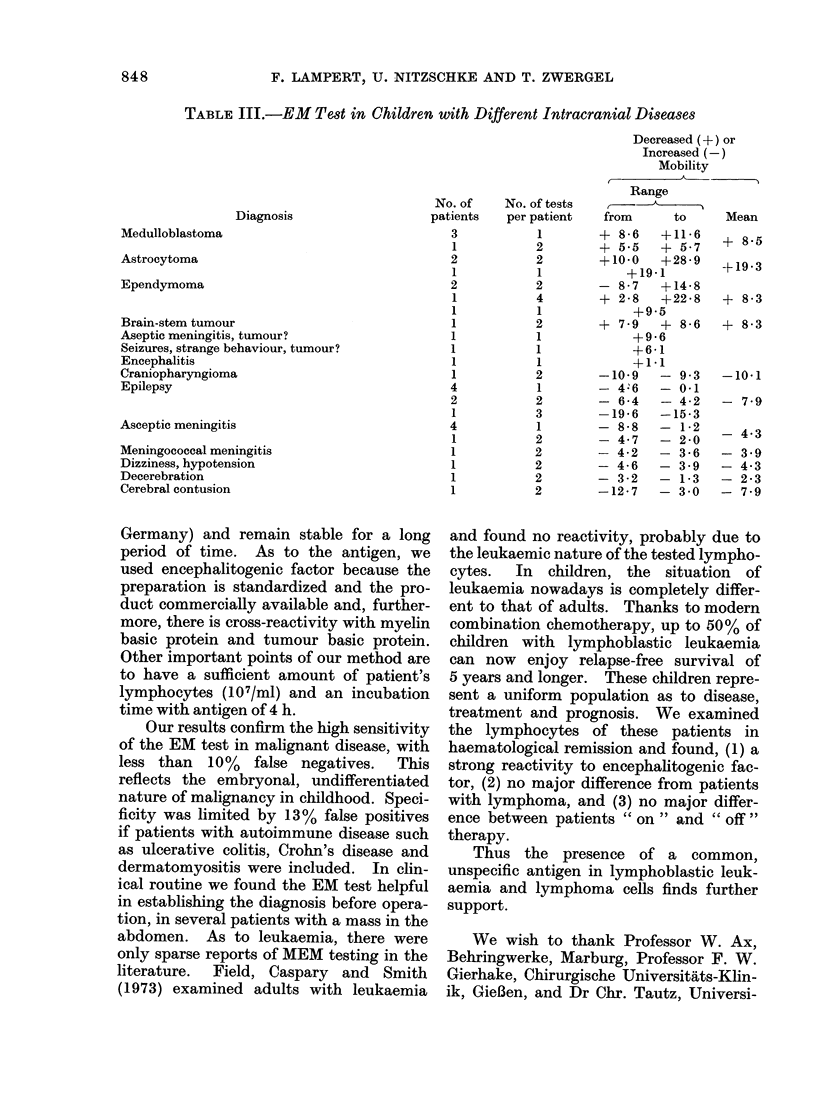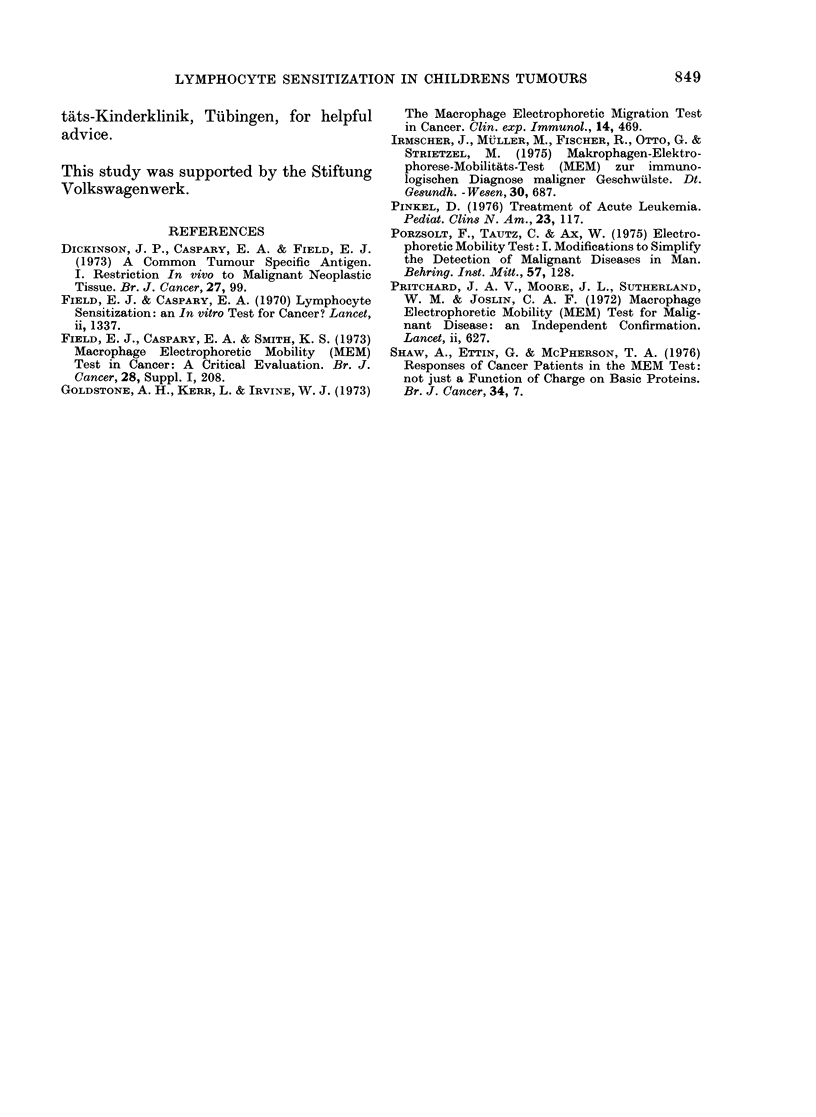# Lymphocyte sensitization in childhood solid tumours and lymphoblastic leukaemia, measured by electrophoretic mobility test.

**DOI:** 10.1038/bjc.1977.127

**Published:** 1977-06

**Authors:** F. Lampert, U. Nitzschke, T. Zwergel

## Abstract

A modified electrophoretic mobility (EM) test was performed in 150 children to examine their lymphocyte sensitization to myelin basic protein (encephalitogenic factor). Measurements in the cytopherometer were facilitated by using devitalized sheep erythrocytes as indicator particles instead of macrophages. A significant decrease in EM was found in 29/30 children with acute lymphoblastic leukaemia and in 67/75 children with solid tumours, thus giving a false negative rate in malignant disease of 9/105=8-6%, as compared to 6 false positives among 45 children with non-malignant disorders; 5 of the later "false/positive" 6 patients had autoimmune disease. Results of the EM test in the children with leukaemia were compared with those in 9 patients with non-Hodgkin's lymphoma and 2 with Hodgkin's disease at different stages, but no striking change was seen between different diseases, or after cessation of long-term immunosuppressive chemotherapy. Percentage of "slowing" ranged from 4 to 30%. These results indicate that patients with lymphoid malignancies still have lymphocytes which had been sensitized by a common antigen of the malignant cell clone at the beginning of the disease. The EM test, furthermore, could serve as an additional diagnostic aid in differentiating benign from malignant masses in the abdomen, extremities or intracranial disease.


*Br. J. Cancer* (1977) **35**, 844.

### LYMPHOCYTE SENSITIZATION IN CHILDHOOD SOLID TUMOURS AND LYMPHOBLASTIC LEUKAEMIA, MEASURED BY ELECTROPHORETIC MOBILITY TEST

F. LAMPERT, U. NITZSCHKE AND T. ZWERGEL

*From the Kinderpoliklinik, Justus Liebig-University, 6300 Giessen, W. Germany*

Received 22 November 1976 Accepted 14 February 1977

**Summary.—A modified electrophoretic mobility (EM) test was performed in 150 children to examine their lymphocyte sensitization to myelin basic protein (encephalitogenic factor). Measurements in the cytopherometer were facilitated by using devitalized sheep erythrocytes as indicator particles instead of macrophages. A significant decrease in EM was found in 29/30 children with acute lymphoblastic leukaemia and in 67/75 children with solid tumours, thus giving a false negative rate in malignant disease of 9/105 = 8·6%, as compared to 6 false positives among 45 children with non-malignant disorders; 5 of the latter "false/positive" 6 patients had autoimmune disease. Results of the EM test in the children with leukaemia were compared with those in 9 patients with non-Hodgkin's lymphoma and 2 with Hodgkin's disease at different stages, but no striking change was seen between different diseases, or after cessation of long-term immunosuppressive chemotherapy. Percentage of "slowing" ranged from 4 to 30%. These results indicate that patients with lymphoid malignancies still have lymphocytes which had been sensitized by a common antigen of the malignant cell clone at the beginning of the disease. The EM test, furthermore, could serve as an additional diagnostic aid in differentiating benign from malignant masses in the abdomen, extremities or intracranial disease.**

IN UTILIZING the macrophage electrophoretic mobility (MEM) test Field and Caspary (1970) found that the peripheral blood lymphocytes of patients with cancer were sensitized to a basic protein derived from human brain (encephalitogenic factor—EF).

Subsequent reports confirmed the validity of the MEM test in distinguishing between patients with benign and malignant diseases (Pritchard *et al.*, 1972; Goldstone, Kerr and Irvine, 1973; Irmscher *et al.*, 1975; Shaw, Ettin and McPherson, 1976).

The electrophoretic mobility (EM) test was recently modified by replacing guinea-pig macrophages by devitalized sheep erythrocytes as indicator particles in the cytopherometer (Porzsolt, Tautz and Ax, 1975). The modification made measurements in the cytopherometer easier and more reliable.

The purpose of our investigation was (1) to apply this modified EM test with EF antigen to children with malignancies, as childhood tumours are clinically and cytologically different from adult cancer disease, and (2) particularly, to test lymphocytes of children with lymphoblastic leukaemia and lymphoma in long-term remission, where no EF sensitization has been previously reported.

### MATERIALS AND METHODS

*Patients.*—Peripheral blood was obtained from 150 patients: 30 children with acute lymphoblastic leukemia (ALL) in haematological remission, 75 children with solid tumours, and 45 children with non-malignant diseases. Twenty-five of the patients (10 with

Address for reprints: Prof. Fritz Lampert, Universitäts-Kinderpoliklinik, Feulgenstraße 12, 6300 Lahn-Gießen 1, West Germany.

ALL + 15 with solid tumours) could be studied at the Children's Hospital, University of Munich, thanks to the generous help of Drs R. J. Haas and R. Eife. Treatment of the patients with ALL consisted of combination chemotherapy for 2 to 3 years, including prophylactic cranial irradiation, leading to a 5-year leukaemia-free survival of 30–50% (Pinkel, 1976).

*Lymphocyte isolation.*—Fifteen to 20 ml of heparinized whole blood (Liquemin® Roche 500 i.u./10 ml) was diluted with equal amounts of Hanks's solution (Flow Laboratories) containing 0·005% EDTA (Merck) and layered over Triosil-Ficoll® in sterile 80-ml glass centrifuge tubes. The lymphocyte-enriched interface was recovered after centrifugation (400 *g*, 30 min) and washed once in Hanks's solution with 0·005% EDTA (400 *g*, 15 min). The cells were then transferred into sterile polypropylene tubes, washed twice in Hanks's balanced salt solution and resuspended in 1 ml of Dulbecco's medium to a final concentration of $10^4$ cells/$\mu$l.

*Antigen preparation.*—The encephalitogenic factor (EF, kindly supplied by Prof. Ax, Behringwerke, Marburg) was prepared as crude EF protein from normal human brain as described by Dickinson, Caspary and Field (1973).

*Incubation procedures.*—Incubations with antigen and indicator particles were performed according to Porzsolt *et al.* (1975): 0·7 ml of the lymphocyte suspension was incubated with 3·0 ml of EF solution (0·3 mg EF/ml Hanks's balanced salt solution, HBSS) for 4 h at 37 °C. The cells were then spun at 800 *g* for 15 min, and 3 ml of the supernatant was pipetted off for either immediate processing or storage at −20 °C. After thawing, 3 ml of the supernatant was incubated with 1 ml of a suspension of tanned and sulphosalicylic-acid-stabilized sheep erythrocytes (ETS, kindly supplied by Prof. Ax, Behringwerke, Marburg) ($5 \times 10^7$ ETS/ml HBSS) for 90 min at 23 °C. As a control, supernatant without EF was prepared for each test, 1 ml ETS ($5 \times 10^7$ ETS/ml HBSS) was incubated with 3 ml of a mixture of Dulbecco's medium and HBSS (7 : 30) for 90 min at 23 °C in a water bath.

*Cytopherometer.*—Measurements of ETS-electrophoretic mobility were performed in a Zeiss cytopherometer, using a current of 6 mA at 110 V, and maintaining the observation chamber at a temperature of 23·0 °C. All samples were presented for blind measurements in random order.

*Measurements and calculations.*—For each incubation mixture, 20 erythrocytes were timed with two stopwatches across the given distance of 16 $\mu$m in the ocular eye-piece in both directions of the potential difference. To prevent timing errors by cell drifting, actual migration time was expressed by the formula $t = 2a(b-a)/b$ (Porzsolt *et al.*, 1975) in which $t$ is the actual migration time, $a$ the migration time in one direction, and $b$ the total migration time in both directions. For each specimen, the mean actual migration time of 20 ETS indicator particles ± the standard deviation was determined in relation to the control which was freshly prepared for each day that measurements were made. The % reduction in mobility was calculated according to the formula:

$$\% \text{ slowing} - \frac{\text{mean of test sample} - \text{mean of control}}{\text{mean of control}} \times 100$$

The results, *i.e.* increased or decreased mobility, were always tested for statistical significance (Student's *t* test) in relation to the control; "Positive" results ( + ) indicate a significant decrease in mobility as compared to the control; "negative" results ( — ) either are not significantly different to the control or show a significant increase in mobility.

Two controls were always freshly prepared for each day of measuring. This reference was necessary as the ETS indicator particles, depending on age or different donor sheep, showed variations in mobility. The timing of all controls was 4·3 ± 0·6 s (mean ± s.d.).

### RESULTS

A scattergram showing the pooled results of 150 children with different malignant and non-malignant disorders is presented in Fig. 1. The data of the 3 groups of Fig. 1 were statistically examined by the Scheffe test. The differences between the group of non-malignant disorders (with or without autoimmune disease) and the groups of malignant tumours and acute lymphoblastic leukaemia were highly significant ($P < 0·001$). No significant difference was seen between

FIG.—Results of electrophoretic mobility (EM) test on 150 children.

the groups of acute lymphoblastic leukaemia and malignant solid tumours. In comparing the results in relation to the control, there were 67/75 children with tumours and 29/30 children with acute lymphoblastic leukaemia (all in haematological remission) who responded to lymphocyte stimulation by EF antigen, *i.e.* mobility inhibition. This gives a total of 9/105 = 8·6% false negatives, or a cancer sensitivity of over 90%. In contrast, almost all 45 children with non-malignant diseases had either significant " negative slowing" or no significant " positive slowing ". Five of 6 false positive results were children with autoimmune disorders (one with rheumatoid arthritis, one with ulcerative colitis, 2 with Crohn's disease, one with dermatomyositis). The other patients in the group of non-malignant diseases had haematological, renal or metabolic disorders, or acute infections. The meaning of increased mobility in these patients remains obscure. Patients with lymphoblastic leukaemia and lymphoma in different stages of their disease were compared in Table I. Only one child with ALL still in remission had a " negative slowing ". All the others had a significant slowing relative to the control, ranging from 4 to 28%. No real major difference was seen either between the different lymphoid malignancies| or between " on " and " off " therapy. " Off therapy patients " had a slowing in the range of 9 to 21%, " on therapy patients " a similar range of slowing (10 to 28%). Non-Hodgkin's lymphoma patients under treatment had a slowing ranging from 7 to 30%.

TABLE I.—*EM Test in Children with Acute Lymphoblastic Leukaemia (ALL), Hodgkin's Disease (HD) and Non-Hodgkin's Lymphoma (NHL)*

| Diagnosis, current status | No. of patients | No. of tests per patient | Decreased (+) or Increased (−) Mobility: Range from | Range to | Mean |
|---|---|---|---|---|---|
| ALL complete remission, off therapy | 5 | 1 | +11·3 | +16·1 | +13·4 |
| | 4 | 2 | + 7·4 | +21·0 | |
| ALL complete remission, on therapy | 17 | 1 | + 7·1 | +27·9 | +16·5 |
| | 1 | 2 | + 9·3 | +18·7 | |
| ALL 2 months after diagnosis | 1 | 2 | + 9·1 | + 9·4 | + 7·4 |
| | 1 | 1 | + 3·7 | | |
| ALL 2¼ years after diagnosis | 1 | 1 | − 2·2 | | |
| HD, Stage II$_A$ in complete remission, off therapy | 1 | 2 | + 4·2 | + 4·7 | + 4·5 |
| HD, Stage II$_B$ in complete remission, on therapy | 1 | 4 | + 5·0 | +22·1 | +10·2 |
| NHL continuous complete remission, off therapy | 1 | 4 | + 8·1 | +25·5 | +15·1 |
| NHL complete remission, on therapy | 3 | 2 | +10·8 | +30·3 | |
| | 3 | 1 | +10·4 | +13·1 | +15·1 |
| | 3 | 3 | + 8·0 | +16·9 | |

Results of the EM test in children with solid tumours of the abdomen and extremities in different stages of their diseases, are summarized in Table II. Only 1/26 patients, a 9-year-old boy whose adrenal neuroblastoma was removed 5 years earlier, had a " negative slowing ". No difference was seen between patients with tumour (*e.g.* before operation) or tumour-free. Greatest slowing was produced by lymphocytes of an 11-year-old girl with malignant teratoma (teratoid chorionepithelioma) which had been removed 6 years earlier.

EM testing in children with intracranial disease (Table III) could clearly distinguish between cerebellar medulloblastoma, astrocytoma and brainstem tumour, giving a " positive slowing ", from epilepsy and aseptic meningitis with " negative slowing ". Range of the 4 patients with ependymoma was from a negative slowing of $-9\%$ to a positive slowing of over $+20\%$. One patient with old excised craniopharyngeoma and diabetes insipidus was strikingly negative ($-10\%$).

### DISCUSSION

Among *in vitro* tests to detect lymphocyte sensitization against tumour-associated antigens (*e.g.* cytotoxicity techniques, macrophage migration inhibition, leucocyte adherence inhibition), the MEM test is credited with the greatest sensitivity. Immunological specificity, however, is somewhat limited, in that not only patients with malignant diseases but also those with autoimmune disorders, as well as with demyelinating disease, give a positive reaction to myelin basic protein or tumour basic protein as antigen.

Universal acceptance of the MEM test as a test for cancer was also hampered by the difficult performance of the cytopherometer recordings.

Most of the technical difficulties of the MEM test, however, can be attributed to the guinea-pig macrophages as indicator cells. These problems have now been overcome by the modification which we adopted from Porzsolt *et al* (1975) of using tanned and sulphosalicylic-acid-stabilized sheep erythrocytes (ETS) instead of peritoneal macrophages. These erythrocytes represent a uniform population in size and migration, can be obtained in large quantities as a standardized commercial product (Tanned and Sulphosalicylic-acid-stabilized Sheep Erythrocytes, ETS, Behringwerke, Marburg, W.

TABLE II.—*EM Test in Children with Malignant Tumours of the Abdomen and Extremities*

| Diagnosis, current status | No. of patients | No. of tests per patient | Decreased (+) or Increased (−) Mobility: Range from | to | Mean |
|---|---|---|---|---|---|
| Neuroblastoma, with tumour | 1 | 1 | +9·2 | | |
| Neuroblastoma, tumour removed | 3 | 2 | +13·7 | +22·1 | |
| | 1 | 3 | + 8·25 | +13·6 | +14·5 |
| | 1 | 1 | + 9·6 | | |
| Neuroblastoma, no tumour for 5 years | 1 | 2 | − 1·9 | − 0·9 | − 1·4 |
| Wilm's tumour, with tumour | 1 | 1 | +16·1 | | +13·2 |
| | 1 | 4 | + 7·8 | +16·5 | |
| Wilm's tumour, tumour removed | 3 | 1 | +11·3 | +19·8 | +14·4 |
| | 2 | 2 | +13·8 | +14·4 | |
| Rhabdomyosarcoma | 2 | 4 | +12·6 | +23·0 | +12·7 |
| | 2 | 1 | +14·0 | +21·0 | |
| Osteosarcoma, after amputation | 2 | 2 | + 5·4 | + 8·7 | + 7·9 |
| | 1 | 1 | +15·8 | | |
| Ewing's sarcoma | 2 | 1 | +12·4 | +15·2 | + 9·8 |
| | 1 | 3 | + 5·6 | +12·8 | |
| Chondrosarcoma, with tumour | 1 | 2 | + 5·8 | +12·2 | + 6·1 |
| Malignant teratoma, tumour removed | 1 | 3 | +25·8 | +31·7 | +28·3 |

TABLE III.—*EM Test in Children with Different Intracranial Diseases*

| | | | Decreased (+) or Increased (−) Mobility | | |
|---|---|---|---|---|---|
| | | | Range | | |
| Diagnosis | No. of patients | No. of tests per patient | from | to | Mean |
| Medulloblastoma | 3 | 1 | + 8·6 | +11·6 | + 8·5 |
| | 1 | 2 | + 5·5 | + 5·7 | |
| Astrocytoma | 2 | 2 | +10·0 | +28·9 | +19·3 |
| | 1 | 1 | +19·1 | | |
| Ependymoma | 2 | 2 | − 8·7 | +14·8 | |
| | 1 | 4 | + 2·8 | +22·8 | + 8·3 |
| | 1 | 1 | +9·5 | | |
| Brain-stem tumour | 1 | 2 | + 7·9 | + 8·6 | + 8·3 |
| Aseptic meningitis, tumour? | 1 | 1 | +9·6 | | |
| Seizures, strange behaviour, tumour? | 1 | 1 | +6·1 | | |
| Encephalitis | 1 | 1 | +1·1 | | |
| Craniopharyngioma | 1 | 2 | −10·9 | − 9·3 | −10·1 |
| Epilepsy | 4 | 1 | − 4·6 | − 0·1 | |
| | 2 | 2 | − 6·4 | − 4·2 | − 7·9 |
| | 1 | 3 | −19·6 | −15·3 | |
| Asceptic meningitis | 4 | 1 | − 8·8 | − 1·2 | − 4·3 |
| | 1 | 2 | − 4·7 | − 2·0 | |
| Meningococcal meningitis | 1 | 2 | − 4·2 | − 3·6 | − 3·9 |
| Dizziness, hypotension | 1 | 2 | − 4·6 | − 3·9 | − 4·3 |
| Decerebration | 1 | 2 | − 3·2 | − 1·3 | − 2·3 |
| Cerebral contusion | 1 | 2 | −12·7 | − 3·0 | − 7·9 |

Germany) and remain stable for a long period of time. As to the antigen, we used encephalitogenic factor because the preparation is standardized and the product commercially available and, furthermore, there is cross-reactivity with myelin basic protein and tumour basic protein. Other important points of our method are to have a sufficient amount of patient's lymphocytes ($10^7$/ml) and an incubation time with antigen of 4 h.

Our results confirm the high sensitivity of the EM test in malignant disease, with less than 10% false negatives. This reflects the embryonal, undifferentiated nature of malignancy in childhood. Specificity was limited by 13% false positives if patients with autoimmune disease such as ulcerative colitis, Crohn's disease and dermatomyositis were included. In clinical routine we found the EM test helpful in establishing the diagnosis before operation, in several patients with a mass in the abdomen. As to leukaemia, there were only sparse reports of MEM testing in the literature. Field, Caspary and Smith (1973) examined adults with leukaemia and found no reactivity, probably due to the leukaemic nature of the tested lymphocytes. In children, the situation of leukaemia nowadays is completely different to that of adults. Thanks to modern combination chemotherapy, up to 50% of children with lymphoblastic leukaemia can now enjoy relapse-free survival of 5 years and longer. These children represent a uniform population as to disease, treatment and prognosis. We examined the lymphocytes of these patients in haematological remission and found, (1) a strong reactivity to encephalitogenic factor, (2) no major difference from patients with lymphoma, and (3) no major difference between patients " on " and " off " therapy.

Thus the presence of a common, unspecific antigen in lymphoblastic leukaemia and lymphoma cells finds further support.

We wish to thank Professor W. Ax, Behringwerke, Marburg, Professor F. W. Gierhake, Chirurgische Universitäts-Klinik, Gießen, and Dr Chr. Tautz, Universitäts-Kinderklinik, Tübingen, for helpful advice.

This study was supported by the Stiftung Volkswagenwerk.

### REFERENCES

DICKINSON, J. P., CASPARY, E. A. & FIELD, E. J. (1973) A Common Tumour Specific Antigen. I. Restriction *In vivo* to Malignant Neoplastic Tissue. *Br. J. Cancer*, **27**, 99.

FIELD, E. J. & CASPARY, E. A. (1970) Lymphocyte Sensitization: an *In vitro* Test for Cancer? *Lancet*, ii, 1337.

FIELD, E. J., CASPARY, E. A. & SMITH, K. S. (1973) Macrophage Electrophoretic Mobility (MEM) Test in Cancer: A Critical Evaluation. *Br. J. Cancer*, **28**, Suppl. I, 208.

GOLDSTONE, A. H., KERR, L. & IRVINE, W. J. (1973) The Macrophage Electrophoretic Migration Test in Cancer. *Clin. exp. Immunol.*, **14**, 469.

IRMSCHER, J., MÜLLER, M., FISCHER, R., OTTO, G. & STRIETZEL, M. (1975) Makrophagen-Elektrophorese-Mobilitäts-Test (MEM) zur immunologischen Diagnose maligner Geschwülste. *Dt. Gesundh. -Wesen*, **30**, 687.

PINKEL, D. (1976) Treatment of Acute Leukemia. *Pediat. Clins N. Am.*, **23**, 117.

PORZSOLT, F., TAUTZ, C. & AX, W. (1975) Electrophoretic Mobility Test: I. Modifications to Simplify the Detection of Malignant Diseases in Man. *Behring. Inst. Mitt.*, **57**, 128.

PRITCHARD, J. A. V., MOORE, J. L., SUTHERLAND, W. M. & JOSLIN, C. A. F. (1972) Macrophage Electrophoretic Mobility (MEM) Test for Malignant Disease: an Independent Confirmation. *Lancet*, ii, 627.

SHAW, A., ETTIN, G. & MCPHERSON, T. A. (1976) Responses of Cancer Patients in the MEM Test: not just a Function of Charge on Basic Proteins. *Br. J. Cancer*, **34**, 7.